# The Goldilocks Effect: Human Infants Allocate Attention to Visual Sequences That Are Neither Too Simple Nor Too Complex

**DOI:** 10.1371/journal.pone.0036399

**Published:** 2012-05-23

**Authors:** Celeste Kidd, Steven T. Piantadosi, Richard N. Aslin

**Affiliations:** 1 Brain and Cognitive Sciences, University of Rochester, Rochester, New York, United States of America; 2 Center for Visual Science, University of Rochester, Rochester, New York, United States of America; University of Barcelona, Spain

## Abstract

Human infants, like immature members of any species, must be highly selective in sampling information from their environment to learn efficiently. Failure to be selective would waste precious computational resources on material that is already known (too simple) or unknowable (too complex). In two experiments with 7- and 8-month-olds, we measure infants’ visual attention to sequences of events varying in complexity, as determined by an ideal learner model. Infants’ probability of looking away was greatest on stimulus items whose complexity (negative log probability) according to the model was either very low or very high. These results suggest a principle of infant attention that may have broad applicability: infants implicitly seek to maintain intermediate rates of information absorption and avoid wasting cognitive resources on overly simple or overly complex events.

## Introduction

Human infants face two daunting problems as they begin to learn about their surroundings. First, they enter the postnatal world with only rudimentary mechanisms–provided by their evolutionary heritage–for interpreting environmental information. Second, the potential information available in the environment is both voluminous and complex. These two problems led William James to coin his famous phrase about “the blooming, buzzing confusion” that confronts the newborn [1]. Nonetheless, infants show remarkable feats of learning, beginning in the last trimester of fetal life, continuing through the perinatal period, and accelerating through infancy and early childhood [2]–[5]. Infants are able to extract the statistical properties of their environment in a diverse array of learning tasks and domains, including sounds, words, people, shapes, and objects [6]–[11]. But how is it that infants are able to learn efficiently in such a complex environment? One solution is to have a small set of innate biases; for example, seeking to look at and listen to biologically significant stimuli such as faces and speech. However, innate biases alone cannot be the solution for the vast majority of stimuli from which infants must learn. Given the slow time-course of evolution, we also need general purpose learning mechanisms to deal with a changing environment and with classes of stimuli that could not plausibly be processed by a small set of specialized mechanisms.

Here, we focus on this general-purpose learning mechanism by avoiding the use of special stimuli and asking whether infants deploy a sensible (and likely implicit) strategy for allocating attention to arbitrary, neutral stimuli. Our goal is to determine whether infants are biased to gather information from the environment in a principled way that serves as a key component of an efficient learning mechanism [12], [13]. Specifically, we provide evidence that infants avoid spending time examining stimuli that are either *too simple* (highly predictable) or *too complex* (highly unexpected) according to their implicit beliefs about the probabilistic structure of events in the world. Rather, infants allocate their greatest amount of attention to events of intermediate surprisingness–events that are likely to have *just enough* complexity so that they are interesting, but not so much that they cannot be understood. This approach builds on a longstanding tradition in developmental psychology, as exemplified by Piaget [13]. He argued that when children are confronted with a new piece of information, they initially attempt to incorporate it within their existing knowledge structures through a process of *assimilation*. When this is not possible, children either fail to learn new structures (and move on to sample other information) or they adapt by creating new knowledge structures, a process he called *accommodation*.

Piaget had no objective measure of assimilation or accommodation; they remained hypothetical constructs. However, in subsequent research, a proxy for these theoretical constructs centered on the relative duration of visual attention to objects or events varying in complexity or familiarity. Many researchers have speculated about what underlying mental operations are indexed by infants’ looking times or attentional patterns [14] (for review, see Aslin 2007 [15]). The generally accepted view is that looking times reflect some combination of (a) stimulus-driven attention, (b) memory of past stimuli, and (c) comparison between the current and the past stimuli. If infants are presented with an already familiar stimulus, they prefer it over a novel stimulus, but quickly tire of it after a brief period of re-familiarization (*habituation*), and subsequently show preferences for novel stimuli. Similarly, if repeatedly exposed to an initially novel stimulus, infant looking times decline and then recover to the presentation of another novel (i.e., completely unfamiliar) stimulus. Theoretical accounts for these familiarity and novelty preferences all share a common theme: As infants attempt to encode various features of a visual stimulus, the efficiency or depth of this encoding process determines their subsequent preferences. Familiarity preferences arise when infants have not yet completed encoding the familiar stimulus into memory, or when the novel stimulus is too dissimilar from the infants’ existing mental representations to be readily encoded [16]–[22].

However, these theories lacked an objective measure of the relevant independent variable–an event’s *complexity* or *relationship to existing representations*. Instead, researchers overwhelmingly relied on qualitative judgments of stimulus complexity to select materials to test infants’ visual preferences. These qualitative judgments relied on inferences about infants’ existing mental representations, to which researchers had no direct access. With no reasonable way of modeling infants’ existing representations, it was impossible to quantitatively measure the complexity of the information conveyed by a particular stimulus. Thus, researchers had only *post hoc* estimates of stimulus complexity–those obtained by measuring the very patterns of visual preferences that the theories were designed to predict. Two exceptions are Civan, Teller & Palmer 2005 [23] and Kaldy & Blaser 2006 [24] in that both papers quantified the perceptual salience of visual stimuli in order to effectively demonstrate its importance in eliciting infants’ preferences for novel versus familiar stimuli.

We overcome these problems by formalizing a notion of stimulus complexity and behaviorally testing the relationship between complexity and infants’ probability of looking away at each successive point in a sequence of events. We assume that at each point in the experiment–and in everyday life–infants have used observed data to form probabilistic expectations about what events are likely and unlikely to be observed next [25], [26]. We model these expectations using an idealized observer model of our experimental stimuli. We then measure complexity as the *negative log probability* of an event according to this idealized model. This measure quantifies each event’s *information content* [27]. (This measure has also been called *surprisal* [28], since it may also be interpreted as representing the “surprise” of seeing the outcome.) We show that infants preferentially look away at events that are either very simple (high probability) or very complex (low probability), according to the idealized model. Intuitively, high probability events convey little information–infants’ attentional resources are best spent elsewhere. Low probability events may indicate that the observed stimuli are unlearnable, unstructured, or difficult to use predictively in the future. Negative log probability also quantifies the number of *bits* of information an ideal observer would require to encode that sequence of events in memory. Thus, infants may avoid stimuli that require encoding too much information or information that could only be extracted by prolonged attention to rare events, thereby incurring a higher processing cost than shifting attention to less complex events.

### Experiment and Modeling Approach

**Figure 1:**
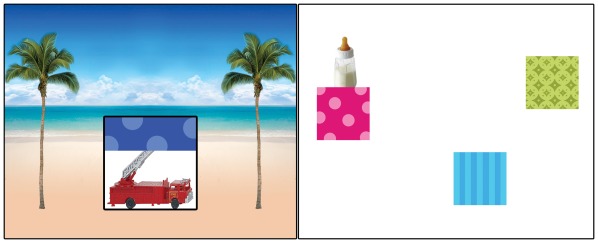
Examples of visual displays used in Experiments 1 and 2. ***a*** **)** The object (e.g., a toy fire truck) in the box for Experiment 1 was revealed (or not) by up-down animation of an occluder (e.g., a blue polka-dotted box). ***b*** **)** In Experiment 2, one of three unique objects (e.g., a baby bottle) popped up from behind one of three highly distinctive boxes. Also see **Videos S1** and **S2** for examples of animated displays used in these studies.

The behavioral experiment measured the point, in a sequence of events, when an infant looked away from a visual display. The displayed stimuli were easily captured by a simple statistical model. In Experiment 1, we presented each infant with 42 unique animated displays, each featuring one of 42 uniquely colored and patterned boxes occluding one of 42 unique familiar objects (e.g., a ball). Each scene display began with the occluder rising and falling, thus appearing to reveal and then re-obscure the object hidden behind it (**Fig. 1(a)** and **Video S1**). To maintain infants’ attention early in the experiment, the first reveal always showed an object. For example, a blue polka-dotted occluder might rise to reveal a toy fire truck. On subsequent reveals, the same object appeared in the box according to some probability randomly assigned to that trial. For example, if a trial were associated with the probability of 0.3, then 30% of the time an object would be present behind the box. Probabilities ranged from 0 to 1 in increments of 0.05 (i.e., 0.0, 0.05, 0.1, 0.15, etc.), such that there were 21 possible probabilities-of-appearance that could be associated with an object on a particular trial. The sequences of object reveals thus varied in terms of their information-theoretic properties: some events in a sequence were highly predictable (e.g., a ball appears still in the box after having appeared on each of ten previous reveals), and others were less predictable (e.g., a rattle appears to have *disappeared* from within the box after having appeared on each of the ten previous reveals). The objects, boxes, and order in which the probabilities-of-appearance were presented were randomized across infants, and each of the 21 probabilities-of-appearance occurred twice (for a total of 42 trials). Each animated sequence of events continued until the infant met the look-away criterion, which was defined as gaze directed off-screen for greater than 1 consecutive second (see **Video S3** for look-away example). To address uncertainty about infants’ mental representations and their age-related or uniquely individual processing speeds and biases for stimulus salience, we exhaustively randomized and counterbalanced all of these extraneous variables (e.g., sequence order, object identity, object familiarity, spatial location).

We modeled the sequences of reveals using a *Markov Dirichlet-multinomial* model (MDM). The Dirichlet-multinomial is a general-purpose statistical model that uses observed event counts to compute a posterior distribution for an underlying multinomial distribution on events. The Dirichlet-multinomial makes parametric assumptions about the form of the prior probability and the likelihood of an event and is often used in Bayesian statistics because of its computational simplicity (see *Materials and Methods*). We apply this to a time-series of events by making a Markov assumption that each event is statistically independent (i.e., not dependent on the ordering of the preceding events). Thus, the model can take some previously observed sequence of events–corresponding to an individual infant’s observations before they have looked away–and compute the probability of every possible next event. We hypothesize that infants’ probability of looking away at the next event in a sequence is at least partially determined by the information-theoretic properties of that event, according to the model. Specifically, at each point in a sequence of events, the model assigns each event a probability, and the negative log of this probability provides a natural information-theoretic measure of the complexity of the next event according to the model’s current expectations about which events are likely.

**Figure 2:**
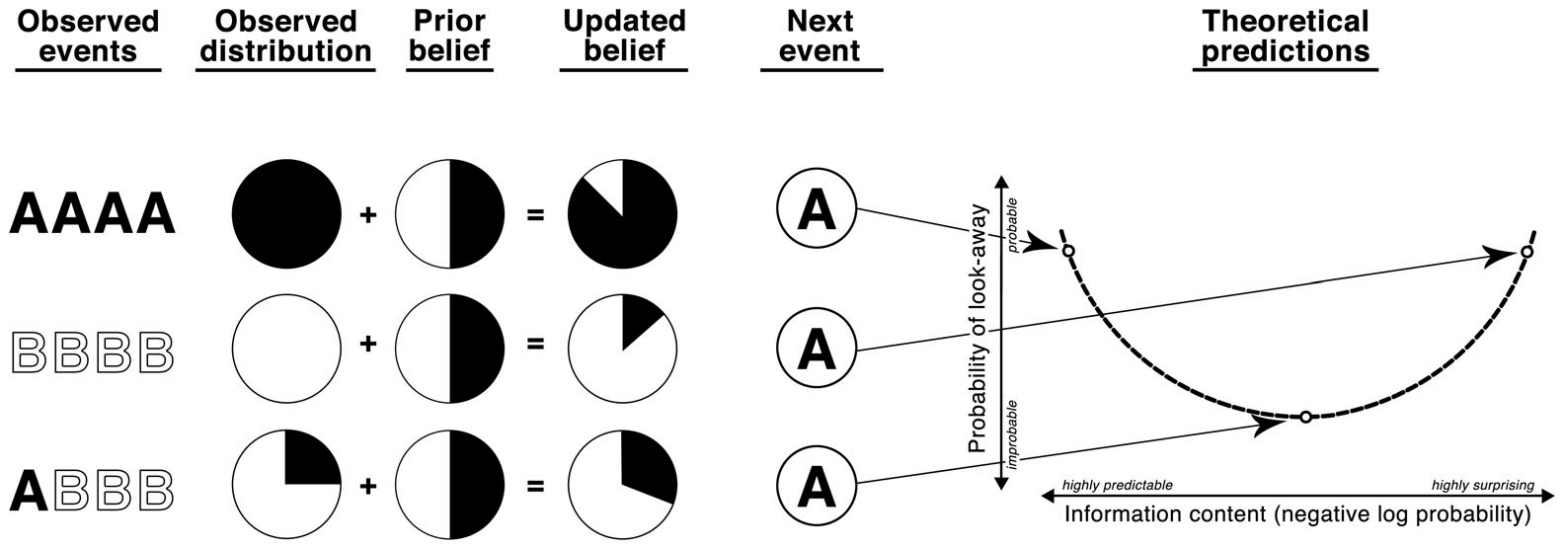
Schematic showing several example event sequences and how the ideal observer model combines observed events with a simple prior to form expectations about upcoming events. The next event then conveys some amount of information according to these probabilistic expectations, which is related to infants’ probability of look-away at a *specific next event* by a U-shaped function.

**** **Fig. 2** illustrates the logic of the experiment and analysis. In the first example, the observer sees a sequence of four *A* events in a row. In this case, the observed data consist of entirely *A*’s. These data are combined with the prior–essentially a smoothing term to avoid zero probabilities–to form an updated posterior belief with high probability of *A* but non-zero probability of *B* (*“Updated belief”* column). The complexity (negative log probability) of the next event is determined using this posterior, which represents the model’s updated belief about the true distribution of events. Thus, if the next event is an *A*–an event that is highly likely according to the model’s posterior–the complexity of that event would be low (i.e., the event would be highly predictable according to the model). We hypothesize that infants would be more likely to look away at this event. Conversely, if the previous observations assign *A* very low probability (second example), *A* will have very high complexity (i.e., the event would be highly surprising according to the model) and infants should terminate the sequence of events by looking away. If the previous observations make *A* moderately likely (third example), the occurrence of an *A* event will convey a “Goldilocks” amount of information, leading infants to be less likely to look away. If infants do not look away, then the modeling step is repeated for the next item in the sequence. This means that infants may look away at different points in different sequences, but we predict systematicity in these look-aways: regardless of how far into a sequence an infant has made it without looking away, their probability of looking away on the next object will depend on its complexity, conditioning on all previous observations.

We note that this type of modeling and analysis contrasts with most previous infant studies, which typically tested for differences in overall mean looking times. Here, we are predicting a binary outcome (whether an infant looks away) at each individual event in the sequence. This is a more precise prediction based on probabilities computed on-line.

## Results

### Experiment 1

**Figure 3:**
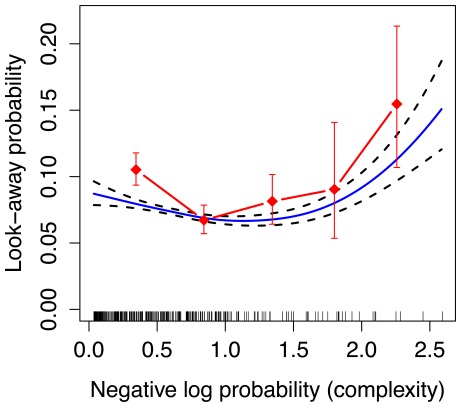
U-shaped curve for single-box display used in Experiment 1. The solid curve represents the fit of a Generalized Additive Model (GAM) [29] with binomial link function, relating complexity according to the MDM model (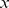-axis) to infants’ look-away probability (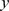-axis). The dashed curves show standard errors according to the GAM. The GAM fits include the effect of complexity (negative log probability) and the effect of position in the sequence. Note, the error bars and GAM errors do not take into account subject effects. Vertical spikes on the 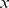-axis represent data points collected at each complexity value. The red diamonds represent the raw look-away probabilities binned along the 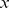-axis.

**Fig. 3** shows infants’ probability of looking away, as a function of that event’s negative log probability according to the model, and collapsing across infants, sequences, and sequence positions. The diamonds show raw probability of look-away, binning complexity into 5 discrete bins. The curve represents the fit of a Generalized Additive Model [29], which attempts to find a smooth relationship between complexity and look-away probability. This figure shows a U-shaped relationship between infant look-away probability and the on-line model-based estimate of complexity, with infants looking away from events that are especially predictable or especially surprising. There is a “Goldilocks” value of complexity around 1.25 bits, corresponding to infants’ preferred information rate in this task.

Although the plot in **Fig. 3** provides a revealing picture of the relationship between indexes of complexity and looking durations, there are likely other factors that influence when infants will look away from the displays. For instance, low-information and high-information events may tend to occur later in a sequence, after learners have developed expectations about the distributional properties of the events. If infants tend not to look away early, perhaps because they are initially captured by the salience of the display independent of its complexity, they would appear to disprefer low and high complexity. To address this potential confound, we performed a regression analysis that controls for the influence of temporal and other factors on look-away probability. When infants look away in a trial, they provide no more data for the remainder of the trial. Because of this, such data violate the independence assumptions of standard logistic (or linear) regression. An appropriate model for this kind of data–used primarily in biostatistics to study, for example, predictors of mortality–is known as a *survival analysis* [30], [31]. We used a type of survival analysis known as a *Cox regression*, that measures the log linear influence of predictors on look-away probability, while respecting the fact that once infants look away they provide no additional data on the same trial. Importantly, this regression also controls for a baseline look-away distribution, which is fit non-parametrically to the data, thereby removing the influence of an average distribution of looking times before testing the significance of the other predictors. We note that this regression does not include subject effects, but we develop more sophisticated analysis methods that include a range of subject effects in forthcoming work [32].

We included a number of control covariates that could plausibly influence infant look-aways using a stepwise procedure that only added variables that improved model fit. These variables included whether an object was present, whether the presence of the object was the same as the previous reveal, how many sequences the infant had already observed, and the uncertainty in the model about the correct distribution of events. This was measured by the differential entropy of the multinomial parameters in the MDM model. We also included linear and quadratic complexity terms. To aid in interpretation of the regression coefficients, complexity was standardized before being squared (i.e., it was shifted and scaled to have mean 0 and standard deviation 1) to test for a significant quadratic trend of complexity on look-aways. This stepwise procedure revealed a significant effect only for squared complexity (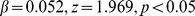), and no other variables (see Table 1). This indicates that the U-shape observed in **Fig. 2** is statistically significant, even after controlling for an overall baseline look-away distribution and the other potentially confounding variables (see *Materials and Methods*). The magnitude of this effect can be understood by considering 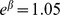, which is the *factor* that the baseline look-away probability is multiplied by for each increase in squared surprisal of 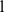 standard deviation from the overall mean in the experiment. This effect is relatively small, though statistically reliable.

**Table 1:** Cox Regression Coefficients.

Covariate	Coefficient	*exp*(coefficient)	Standard error	Z-statistic	P-value
Experiment 1					
Squared complexity	0.052	1.05	0.026	1.969	0.049
Experiment 2 - Non-transitional model					
Complexity	−0.216	0.805	0.094	−2.29	0.022
Squared complexity	0.269	1.308	0.109	2.47	0.013
Trial number	0.029	1.030	0.007	3.99	6.5.10^–5^
Model uncertainty	0.261	1.298	0.174	1.50	0.13
Experiment 2 - Transitional model					
Squared complexity	0.356	1.43	0.084	4.27	1.9.10^–5^
Trial number	0.027	1.03	0.007	3.65	2.7.10^–4^
First appearance	0.500	1.64	0.272	1.82	0.069

All variables found in Experiments 1 and 2 that were added by the stepwise procedure. Note that some non-significant variables are added because the stepwise comparison is based on the Akaike information criterion [40]. These results reveal significant quadratic effects of complexity in both experiments. Complexity and squared complexity were shifted and scaled to have mean of 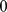 and standard deviation of 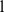 before being entered into the regression.

### Experiment 2

In Experiment 1, objects were either present or absent from behind a single occluder. Perhaps a more typical context in real life, though, is for different events to occur in a multi-object scene, thereby allowing infants’ attention to be attracted to both individual events and transitions between events. In Experiment 2, we presented each infant with 32 unique sequential-event displays (**Fig. 1(b)** and **Video S2**). Each display presented an animated scene consisting of three uniquely patterned boxes, each concealing a unique familiar object (e.g., a cookie). The locations of the three boxes for a given sequence were chosen randomly but remained static throughout a scene. The box locations were randomly shuffled between event sequences, but no more than two boxes appeared on either half of the screen. Neither the patterns on the boxes nor the objects were repeated across event sequences so that each object-box pair was independent and unique. Each event in a sequence consisted of an object that popped out of a box, and then back into the box. Each event lasted 2 seconds in total duration (1-second “pop-up”, 1-second “pop-down”). Events were presented sequentially with no overlap or delay. The same 32 event sequences were presented to every infant. However, the objects, boxes, and order in which the 32 event sequences were presented were randomized across infants. This design ensured that differences in looking times across event sequences were not driven by differences in scene items or presentation order. Each animated sequence of events continued until the infant met the look-away criterion, which was defined as gaze directed off-screen for greater than 1 consecutive second.

**Figure 4:**
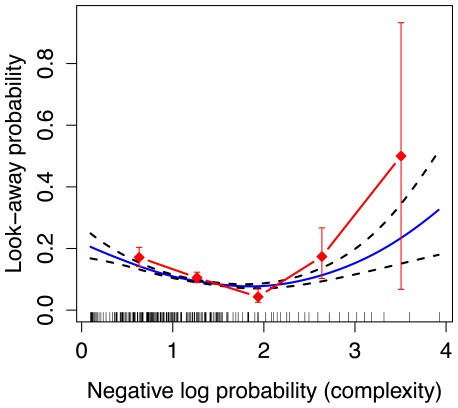
U-shaped curve for three-box display used in Experiment 2. The solid curve represents the fit of a GAM, relating complexity as measured by the *non-transitional* MDM (assuming event independence) to look-away probability. Dashed curves show GAM standard errors. The GAM fits include the effect of complexity (negative log probability) and the effect of position in the sequence. Note, the error bars and GAM errors do not take into account subject effects. Vertical spikes on the 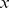-axis represent data points collected at each complexity value. The red diamonds represent the raw look-away probabilities binned along the 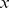-axis.

Results from Experiment 2 are shown in **Fig. 4**. As in Experiment 1, there is a U-shaped relationship between look-away probability and complexity, as measured by the same MDM model (assuming event independence) used in Experiment 1. The Cox regression for Experiment 2 included all of the covariates used in Experiment 1, except whether an object was present, since there was always an object popping up from behind one of the three boxes. However, because there are three different box-object pairs in each scene, we also included covariates measuring whether the current event is the first time an object has appeared from behind a box, and a factor measuring how many objects have not yet popped up. Results of this analysis are shown in Table 1. As in Experiment 1, this analysis revealed significant effects of squared complexity (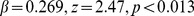). Here, 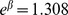, meaning that each increase of squared complexity 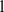 standard deviation from the mean resulted in a look-away probability that was a factor of 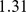 times greater. This is a much larger effect than that found in Experiment 1. There was also a significant linear effect of complexity, indicating that the U is not symmetric about the mean (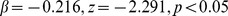), and an effect of trial number, likely representing effects of fatigue (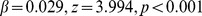), although this is small compared to the complexity effects (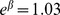).

**Figure 5:**
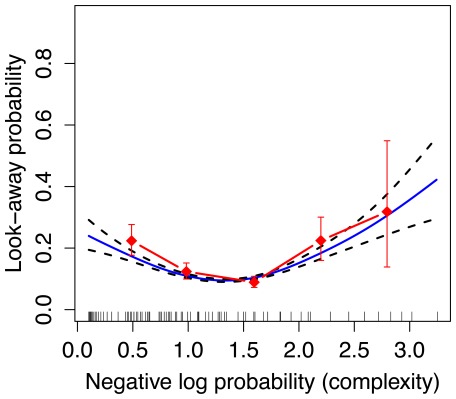
U-shaped curve for three-box display used in Experiment 2. The solid curve represents the fit of a GAM, relating complexity as measured by the *transitional* MDM to look-away probability. Dashed curves show GAM standard errors. The GAM fits include the effect of complexity (negative log probability) and the effect of position in the sequence. Note, the error bars and GAM errors do not take into account subject effects. Vertical spikes on the 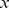-axis represent data points collected at each complexity value. The red diamonds represent the raw look-away probabilities binned along the 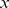-axis.

We also applied the MDM model to the data from Experiment 2 under an assumption of event-order dependence. That is, instead of treating every event as independent, we examined whether look-aways were predicted by the immediately preceding event (i.e., a transitional model). **Fig. 5** shows that a U-shaped function also describes this transitional model, and the Cox regression confirms that this effect is highly significant (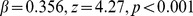). This analysis also revealed an effect of trial-number (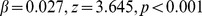).

Finally, one can ask which of the two models better accounts for infants’ behavior on the task in Experiment 2. The predictions of the transitional and non-transitional models are difficult to distinguish because they are closely related: Complexity of both models is correlated at 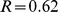 (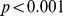). However, if both are entered into a Cox regression along with all variables found to be significant, the transitional complexity is significant (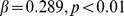), but the non-transitional complexity is not (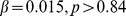). This provides strong evidence that infants track transitional probabilities, but the null result for the non-transitional model is difficult to interpret due to its correlation with the transitional model and the noise inherent in infant data.

## Discussion

The results of the experiments reported here have important implications for two interrelated hypotheses concerning infants’ attention. First, infants behave as if they are employing a principled inferential process for learning about events in the world. The particular MDM model used in our analyses took as inputs a series of observed events or transitions between events to form probabilistic expectations about what events are most likely to occur in the future. The model was necessary to determine what complexity a set of stimulus events conveys to an ideal observer. A failure of either of these components–the probabilistic model or the linking assumption that maps level of complexity onto looking times–would have yielded null results.

Second, infants appear to allocate their attention in order to maintain an intermediate level of complexity. A powerful feature of our analyses was the ability, via the Cox regression, to control for potential confounds such as the number of items that have not appeared yet, item repeats, and an arbitrary baseline distribution of look-away probabilities. To our knowledge, the hypothesis that infants prefer a particular level of information has not been tested while controlling for these other variables, and our analyses therefore provide several methodological advances. Rather than predicting infants’ average looking time to a stimulus, our analyses predicted the precise event in a sequence when an infant would terminate (i.e., look away from) the display. Although others have observed U-shaped behavior in infants under some circumstances, our results provide the first evidence that the information-theoretic properties of a formal model provide a significant predictor of infant look-aways, over and above the effects of other variables, for a large set of arbitrary, neutral visual stimuli. Interestingly, this U-shaped pattern is similar to those obtained with many earlier models of visual attention based on depth of processing or difficulty of encoding the stimulus [17]–[19]. This could indicate that while earlier models did not computationally define the stimulus properties they hypothesized as the mediators of infant looking times (i.e., *complexity*), the properties they explored are nevertheless relevant in guiding infants’ visual attention. Our results also provide a formal account for why infants show novelty preferences (when two test stimuli fall on the left half of the U-shaped function, the stimulus with greater complexity elicits more attention) or familiarity preferences (when two test stimuli fall on the right half of the U-shaped function, the stimulus with lesser complexity elicits more attention).

Similar hypotheses about how adults allocate their limited resources in the language domain–for example, those supporting a uniform information principle [33]–[37]–may suggest that what we have observed in infants reflects a ubiquitous constraint across domains and developmental levels. In addition, other theories propose that learners allocate attention to stimuli containing just the right level of complexity because optimal complexity triggers just the right amount of “*arousal*” in the learner [38]. The U-shaped function may result from the basic response properties of neural systems [39], although determining the precise mechanism will require further research.

In summary, our findings are consistent with theories that suggest infants actively seek to maintain an intermediate level of information absorption, avoiding allocating cognitive resources to either overly predictable or overly surprising events. It is important to note that we are not claiming that this Goldilocks effect is the only factor in infants’ allocation of attention. Certainly, there are species-typical preferences and effects of learning that can dominate infants’ attentional behavior. We argue that when these other factors are controlled for, there remains a significant U-shaped effect of complexity that is well accounted for by our model. Further investigation is required to determine how infants’ preference for intermediate levels of information affects the outcome of learning, either by enhancing the rate of learning or its asymptotic level.

## Materials and Methods

### Ethics Statement

All research was approved by the Research Subjects Review Board at the University of Rochester (protocol RSRB00024570). Parents volunteering their infants for the study were fully informed of the study procedures and completed written informed consent and permission forms in advance of the study.

### Visual Stimuli

In Experiments 1 and 2, we presented infants with animated displays depicting event sequences varying in their predictability. All displays featured uniquely colored and patterned boxes (e.g., pink polka dots) that were animated to reveal unique familiar objects (e.g., a ball; see **Videos S1** and **S2** for examples). A Matlab script was used to generate each of the animated displays. Neither the boxes nor the objects were repeated across event sequences so that each object-box pair was independent and unique. The objects, boxes, and order in which the event sequences were presented were also randomized across infants. This design ensured that differences in looking time across event sequences were not driven by differences in scene items or presentation order.

In Experiment 1, each animated sequence featured *one unique object* occluded by *one box*. The box opened (1 second) and closed (1 second) repeatedly, each time revealing the contents of the box. The object always appeared in the box on the first reveal event. On subsequent reveal events, the object was either present or absent depending on the predictability of the event sequence selected for that trial (a value between 0 and 1). So, for example, a single trial might feature a purple striped box occluding a small toy train with a probability-of-appearance of 0.5. The sequence of events (object appears  = *1*, empty box  = *0*) might be: *1*, *1*, *0*, *1*, *0*, *1*, *0*, *1*, *0*, *0*. The reveals were presented sequentially with no overlap or delay. There were 21 unique probabilities-of-appearance (increments of 0.05 between 0 and 1, e.g., 0, 0.05, 0.1, 0.15, …) and all were presented to each infant twice (42 trials in total) in a random order.

In Experiment 2, each animated display featured *three boxes* of three unique colors and patterns (e.g., yellow stripes, blue polka dots, green stars), *each* concealing a unique object (e.g., a cookie, a spoon, a car). The locations of the three boxes for a given sequence were chosen randomly but remained static throughout a scene. The box locations were randomly shuffled on the screen between event sequences, with the constraint that no more than two boxes appeared on either half of the screen. Each event in a sequence consisted of one of the three unique objects popping out from behind one of the three boxes (1 second), and then back into the box (1 second). Thus, the total duration of each event was 2 seconds, and events were presented sequentially with no overlap or delay. There were 32 unique event sequences that varied in the probability that each of the three objects appeared from behind their respective occluding boxes. Some sequences were simple (e.g., *A*, *A*, *A*, *A*, *A*, *A*, …), while others were more complex (e.g., *A*, *B*, *A*, *B*, *A*, *C*, …). All event sequences were presented to each infant (32 trials in total).

## Methods

The procedures for Experiments 1 and 2 were identical, with two exceptions: the type of displays used (single-box in Experiment 1 and three-box in Experiment 2) and the number of trials presented to each infant (42 in Experiment 1 and 32 in Experiment 2). Each infant was seated on his or her parent’s lap in front of a table-mounted Tobii 1750 eye-tracker. The infant was positioned such that his or her eyes were approximately 23 inches from the monitor, the recommended distance for accurate eye-tracking. At this viewing distance, the 17-inch LCD screen subtended 24×32 degrees of visual angle. Each of the three boxes was 5×5 degrees. To prevent parental influence on the infant’s behavior, the parent holding the infant was asked to wear headphones playing music, wear a visor, lower their eyes, and abstain from interacting with their infant throughout the experiment.

Each trial was preceded by an animation designed to attract the infant’s attention to the center of the screen–a laughing and cooing baby. Once the infant looked at the attention-getter, an experimenter who was observing remotely pushed a button to start the trial. For each trial, an animated scene–featuring a single box in Experiment 1, or three boxes in Experiment 2–was played. The animated sequences of reveal events continued until the infant looked away continuously for 1 second, or until the sequence timed out at 60 seconds. The 1-second look-away criterion for trial termination was automatically determined by the Tobii eye-tracking software. If the infant looked continuously for the entire 60-second sequence, the trial was automatically labeled as a “*time out*” and discarded before the analysis (2.4% of trials in Experiment 1, 5.4% of trials in Experiment 2). If the trial was terminated before the infant actually looked away, the trial was labeled by an experimenter as a “*false stop*” and also discarded. False stops, as determined by a separate video recording of the infant’s face, occurred as a result of the Tobii software being unable to detect the infant’s eyes continuously for 1 second, usually due to the infant inadvertently blocking the eye-tracker camera’s view of his or her own eyes with head or arm movements (22.1% of trials in Experiment 1, and 20.7% of trials in Experiment 2). Trials in which the infant looked for fewer than four events were also discarded, since it is presumed that too few observations are insufficient for establishing expectations about the distribution of events.

### Subjects

In Experiment 1, 42 infants (*mean*  = 7.9 months, *range*  = 7.0 - 8.9) were included in the analysis. Forty-four infants were tested; one infant was excluded due to excessive tiredness (he fell asleep within the first few trials and could not be awakened), and one was excluded due to fussiness. In Experiment 2, 30 infants (mean  = 7.6 months, range  = 7.0 - 8.8) were tested, and all participating infants completed the study. In both studies, all infants were born full-term and had no known health conditions, hearing loss, or visual deficits according to parental report.

### Ideal Learner Model

Intuitively, infants observe how many times each event occurs in the world, and then use these event counts to infer an underlying probability model of their observations. In Experiment 1, the two possible events are that the screen lifts to reveal that an object is either present or absent. In Experiment 2, there are three possible events corresponding to which of three objects appears from behind its box.

An observer who sees only a single event happen would not likely infer that the single observed event is the only one possible (i.e, has probability of 1); instead, observers likely bring expectations to this learning task. In the MDM model used here, this prior expectation is parameterized by a single free parameter, 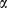, which controls the strength of the learner’s prior belief that the distribution of events is uniform. As 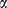 gets large, the model has strong prior beliefs that the distribution of events in the world is uniform; as 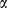 approaches zero, the model believes more strongly that the true distribution closely resembles that of the empirically observed event counts. In modeling, we chose a value of 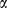 = 1, corresponding to a uniform prior expectation about the distribution of events (with expected values 50-50 in Experiment 1 and 33-33-33 in Experiment 2). However, the qualitative results–in particular, the U-shaped relationship between complexity and look-away probability–do not depend strongly on the choice of 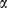.

Formally, suppose there are 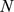 events, 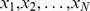, and the 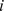
th event has been observed 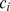 times. We are interested in estimating (or *scoring*) a multinomial distribution parameterized by 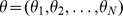 where 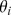 is the true (unobserved) probability of event 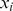. Under a Dirichlet-Multinomial model,
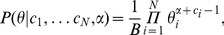
(1)where 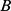 is a normalizing constant that depends on the 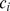 and 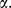 That is, after observing each event type occur some number of times, the infant may form a representation, 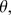 of their guess at the true distribution of events. Every distribution can be scored according to Equation 1, allowing one to compute how strongly a learner should believe that any particular 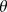 is the correct one. We predict that infants’ likelihood of looking away at a current event will depend upon the complexity of that current event, which is determined by both the previously observed events and the identity of the current event. We predict that events of either very low complexity (highly predictable) or very high complexity (highly surprising) will be more likely to trigger a look-away than events with moderate complexity.

When the 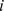
th event occurs, the main variable of interest here is its negative log probability according to the model. We compute this by integrating over the above posterior distribution on 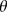. This corresponds to a measure of the information conveyed by observing event 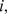 according to an ideal Bayesian learner who had seen all previous events. We predicted that infants would be more likely to look away during events that contained either too little or too much information, giving a U-shaped (quadratic) relationship between this negative log probability measure and the actual observed look-away probability.

## Supporting Information

Video S1 An example of an animated single-box display used in Experiment 1.
(MOV)

Video S2 An example of an animated three-box display used in Experiment 2.
(MOV)

Video S3 A 7-month-old subject attending to a three-box display in Experiment 2, and then looking away (and subsequently terminating the trial).
(MOV)
